# Evaluation of the high definition field of view option of a large-bore computed tomography scanner for radiation therapy simulation

**DOI:** 10.1016/j.phro.2020.03.004

**Published:** 2020-03-26

**Authors:** Richard Y. Wu, Tyler D. Williamson, Narayan Sahoo, Trang Nguyen, Shane M. Ikner, Amy Y. Liu, Paul G. Wisdom, MingFu Lii, Rachel A. Hunter, Paola E. Alvarez, G. Brandon Gunn, Steven J. Frank, Yoshifumi Hojo, X. Ronald Zhu, Michael T. Gillin

**Affiliations:** a Departments of Radiation Physics, The University of Texas MD Anderson Cancer Center, Houston, TX, United States; b Departments of Radiation Oncology, The University of Texas MD Anderson Cancer Center, Houston, TX, United States; c Departments of Imaging and Radiation Oncology Core, The University of Texas MD Anderson Cancer Center, Houston, TX, United States

**Keywords:** TPS, treatment planning system, TLD, thermoluminescent dosimeter, IROC, Imaging and Radiation Oncology Core, PTV, planning target volume, OAR, organ at risk, HD, high-definition option, sFOV, standard field of view option, CT extended field of view, CT number accuracy, Large bore CT scanner, Dose uncertainty

## Abstract

**Background and purpose:**

Computed tomography (CT) scanning is the basis for radiation treatment planning, but the 50-cm standard scanning field of view (sFOV) may be too small for imaging larger patients. We evaluated the 65-cm high-definition (HD) FOV of a large-bore CT scanner for CT number accuracy, geometric distortion, image quality degradation, and dosimetric accuracy of photon treatment plans.

**Materials and methods:**

CT number accuracy was tested by placing two 16-cm acrylic phantoms on either side of a 40-cm phantom to simulate a large patient extending beyond the 50-cm-diameter standard scanning FOV. Dosimetric accuracy was tested using anthropomorphic pelvis and thorax phantoms, with additional acrylic body parts on either side of the phantoms. Two volumetric modulated arc therapy beams (a 15-MV and a 6-MV) were used to cover the planning target volumes. Two-dimensional dose distributions were evaluated with GAFChromic film and point dose accuracy was checked with multiple thermoluminescent dosimeter (TLD) capsules placed in the phantoms. Image quality was tested by placing an American College of Radiology accreditation phantom inside the 40-cm phantom.

**Results:**

The HD FOV showed substantial changes in CT numbers, with differences of 314 HU–725 HU at different density levels. The volume of the body parts extending into the HD FOV was distorted. However, TLD-reported doses for all PTVs agreed within ±3%. Dose agreement in organs at risk were within the passing criteria, and the gamma index pass rate was >97%. Image quality was degraded.

**Conclusions:**

The HD FOV option is adequate for RT simulation and met accreditation standards, although care should be taken during contouring because of reduced image quality.

## 1 Introduction

Computed tomography (CT) is the main imaging modality used for radiation treatment simulation [1], [2]. The 3D CT images are used in the treatment planning systems to calculate the radiation dose to be delivered to the target volumes. However, the standard scanning field of view (sFOV), typically 50 cm in diameter, is much smaller than the bore size, leading to truncation of images and potentially simulation errors for larger patients whose bodies extend beyond the sFOV. One group found that 28% of treated patients did not fit within a 50-cm sFOV CT scanner [3]. A larger FOV imaging mode is needed so that simulation images can be obtained that encompass the patient’s body and any immobilization devices.

Several approaches have been taken to create an extended field of view (eFOV) that is larger than the conventional 50-cm sFOV. Some manufacturers have built CT scanners with large bores (>70-cm diameter); others have designed CT scanners with larger sFOV [4], thereby simplifying patient setup and ensuring that treatment planning images fully capture most patient geometries. Other manufacturers have addressed the problem of image truncation by developing algorithms that reconstruct CT images from a partial dataset at the eFOV [5]. One manufacturer implemented an image reconstruction algorithm in the CT scanner that offers CT number accuracy of ±50 Hounsfield units (HU) in the region between 50 cm and 65 cm (the “high-definition” [HD] FOV) [6] and from 65 cm up to 78 cm (the eFOV), to allow visualization of anatomic structures outside the sFOV.

Significant variation in both CT number accuracy and image distortion have been reported as a function of phantom displacement outside the sFOV [3], [7], [8], which could affect the dosimetric accuracy of the treatment plan created with these images. The extent and impact of dose discrepancies vary among systems, and the uncertainties involved have made some users reluctant to use HD FOV or eFOV.

The effects of using HD FOV or eFOV mode on the dosimetric accuracy of treatment plans have been evaluated by several groups. One study involved shifting the phantom laterally such that a portion of the phantom extends into the eFOV [7]. However, these types of simulation do not accurately represent large patients or their positioning, because patients are normally positioned centrally on the simulation couch, and both sides of their bodies would extend into the HD FOV. Another study evaluated the dosimetric effects of using CT HD FOV [6] with a large-body phantom that extended into the HD FOV region. However, that study did not involve cumulative measurements of dose in a real phantom to confirm the results. Moreover, because CT image reconstruction algorithms and their implementation vary between systems, the truncation effect varies as well. The corresponding reduction of image quality in HD FOV mode has not been quantified. To bridge this gap, we evaluated use of the HD FOV in a large-bore CT scanner for CT number accuracy, geometric image distortion, image degradation and resolution, and dosimetric accuracy of photon treatment planning.

## 2 Materials and methods

### 2.1 CT scanner and image acquisition

A large-bore Siemens SOMATOM Definition Edge CT scanner with a 78-cm gantry aperture and 50-cm sFOV was used for these tests. CT datasets can be reconstructed into an sFOV and image sets of two other sizes, one 65 cm in diameter (the HD FOV) and the other 78 cm in diameter (the eFOV) (Suppl. Fig. S1). Scanning in the HD FOV or eFOV mode allows visualization of anatomy that lies beyond the conventional 50-cm sFOV. The phantoms used for testing were scanned by using an established abdominal CT protocol (120 kVp, 128 × 0.625 mm, 2.0-mm slice thickness, and 300 effective mAs) and images were reconstructed for the sFOV, HD FOV, and eFOV. The treatment planning system supplied dosimetric data in heterogeneous media with the help of tissue characterization based on CT number. The important material property to be considered for photon dose is the mass density [9], [10]. Measurements of CT number vs. mass density using RMI phantom plugs (Gammex, Inc.), with density relative to water ranging from 0 to 1.82, were obtained at the center of the sFOV, between 50 cm and 65 cm (HD FOV), and between 65 cm and 78 cm (eFOV) (Suppl. Fig. S1).

### 2.2 In-House phantoms for evaluating CT number accuracy and image quality

In-house acrylic phantoms (density 1.18 g/cm^3^, thickness 16 cm) were manufactured in one of two sizes: a large (40-cm-diameter) “body” phantom and a small (16-cm-diameter) “extremity” phantom. Two of the smaller phantoms were placed on either side of the large phantom to simulate a large patient whose body extends beyond the sFOV. The large phantom was constructed with a detachable center, with an inner diameter of 20 cm; that hole can be filled with either a 20-cm-diameter acrylic phantom or a standard CT phantom [11] from the American College of Radiology (ACR) for evaluating image quality. The ACR phantom includes inserts to test high- and low-contrast resolution on images. Images were viewed under the ACR-recommended standard viewing conditions (i.e., window = 100 and level = 1100 for high-contrast objects; window = 100 and level = 100 for low-contrast objects). A small hole at the center of the each acrylic phantom (inner diameter 14 mm) allows insertion of RMI mass density plugs with no air gap. The phantoms were designed such that when they are aligned for CT scanning, the mass density plugs at the center of the phantom were located at the center of the sFOV, the center between 50 cm and 65 cm (HD FOV, horizontal distance from imaging center = 28 cm), and the center between 65 cm and 78 cm (eFOV, horizontal distance from imaging center = 37.5 cm) (Suppl. Fig S2a). To test image quality, the ACR CT phantom was placed inside the large-body phantom (Suppl. Fig S2b). The large phantom was first scanned in sFOV mode, and the scan was then repeated with two small acrylic phantoms placed on either side of the large phantom to simulate body parts extending into the HD FOV and the eFOV (Suppl. Fig. S2c).

### 2.3 Anthropomorphic and body-extension-part phantoms

To fully test the dosimetric accuracy of treatment plans for a real patient, we used two anthropomorphic phantoms from the Imaging and Radiation Oncology Core (IROC; Houston, TX)—a pelvis and a thorax—used for tests of dosimetry accuracy and for credentialing radiation oncology sites participating in NCI-sponsored clinical trials [12], [13], [14], [15]. The pelvis phantom includes a 5-cm-diameter sphere that represents the clinical target volume (CTV) (prostate) and critical normal structures (bladder and rectum) (Suppl. Fig. S3a). The phantom contains thermoluminescent dosimeters (TLDs) close to the center of the target and perpendicular holders for GAFChromic films (EBT2) placed in the sagittal and coronal planes, passing through the center of the target. This setup facilitates evaluation of the dose to the center of the prostate as well as analysis of dose distribution. Two other TLDs evaluate the dose to each femoral head. The thoracic phantom also contains an insert that represents part of the left lung, with a centrally located, 3 cm × 5 cm clinical target volume (CTV) (Suppl. Fig. S3b). In this phantom, three orthogonal sheets of GAFChromic film pass through the center of the target, and two TLD capsules are placed within 0.5 cm of the center of the target. The phantom also contains normal structures: the right lung, the heart and the spinal cord, each with a TLD capsule at its center. Schematics of the phantoms showing the position of the TLDs and the films are shown in Supplementary Fig. S4a. Upon receipt of an irradiation phantom, the IROC analyzes the TLDs and radiochromic film [13]. The TLDs are used to evaluate the absolute dose delivered to the phantom. As with the TLDs, the radiochromic film is used for dose profiles through the centers of primary PTVs [14], [15], [16].

We further constructed acrylic body-extension parts to be placed on either side of the anthropomorphic phantoms to simulate large patients (Suppl. Fig. S3c–f). Two surface fiducials, also called BBs, were placed on the apex of each extension part to define the distance between the two extension parts. With the extension parts added, the absolute physical distance between the two BBs was 60 cm. To evaluate the magnitude of phantom image distortion in the HD FOV region, the extended body phantom parts were tied together and placed at the center of the CT couch and imaged in sFOV mode. In this way, the image without distortion can be used as a baseline against which to quantify changes in both volume and CT numbers of the acrylic parts (Suppl. Fig. S3g). The extended body phantom parts, tied together, are 24.5 cm in diameter and 15 cm wide. We used the “body search” tool from the treatment planning system to define regions of interest in the body extension parts.

### 2.4 Treatment planning and phantom irradiation

The Eclipse treatment planning system version 13.7 (Varian Medical Systems, Palo Alto, CA) was used. CT images of the pelvis and thorax anthropomorphic phantoms with the acrylic body-part extensions attached were acquired in HD FOV mode. Body contours with a predefined threshold of –350 HU were automatically created with the Eclipse body searching tool. Contours of TLDs, femoral heads, bladder, prostate and rectum were delineated for the pelvis phantom images. Similarly, contours of TLDs, lungs, heart, and spinal cord were delineated from the thorax phantom images. For both phantoms, the PTVs were created by adding a 0.5-cm margin in the axial plane and a 1-cm margin in the longitudinal plane of the CTV. For the pelvis phantom, a treatment plan was designed with two volumetric modulated arc therapy (VMAT) beams with 15 MV and offset collimator angles: Arc1 (Gantry 210–150, collimator 15) and Arc2 (Gantry 150–210, collimator 345). A total dose of 6 Gy to 98% of the PTV, with maximum dose not to exceed 6.4 Gy, was prescribed. For the thorax phantom, a treatment plan was designed with two VMAT 6-MV arc beams and offset collimator angles: Arc1 (Gantry 181–179, collimator 345) and Arc2 (Gantry 179–181, collimator 15). A total dose of 6 Gy to 95% of the PTV was prescribed. Planning constraints for the normal structures were those used in the IROC phantom planning guidelines (Suppl. Table S1). All dose calculations in this study were derived from the same data table (mass density vs. CT number of the sFOV). Radiation was delivered with a Varian Truebeam system to the pelvis and thorax anthropomorphic phantoms, and the irradiated TLDs and films were sent to IROC for standard processing per their credentialing guidelines [13], [17], [18].

## 3 Results

### 3.1 Variations in CT number

**Fig 1:**
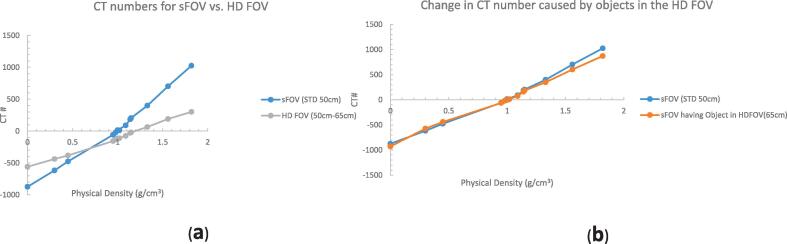
Measurements of CT number vs. mass density using RMI phantom plugs (Gammex, Inc.), with density relative to water ranging from 0 to 1.82, were obtained at the center of the standard field of view (sFOV) and between 50 cm and 65 cm (the high-definition [HD] FOV). (a) Substantial changes in CT numbers were evident on the HD FOV (50 cm to 65 cm). These changes were smaller in dense areas of water or soft tissue and more pronounced on low-density (lung) and high-density (bone) areas. (b) Changes in CT number (measured at the center of the sFOV) caused by body parts extending into the HD FOV and beyond were minimal. Aside from cortical bone, all measurement data from the RMI inserts were within ±100 Hounsfield units.

Differences in CT numbers in the HD FOV region (50 cm to 65 cm) ranged from 314 HU (density 0.0 g/cm^3^) to 725 HU (density 1.82 g/cm^3^) relative to those measured in the sFOV (Fig. 1a). The differences were smaller at the density of water or soft tissue. Smaller changes in CT numbers were apparent for measurements at the center of the image when body parts are extended beyond sFOV and into HD FOV and eFOV region (Fig. 1b). Aside from cortical bone, all of the measurement data from the RMI mass-density plugs were within ±100 HU. Because CT numbers in the eFOV region were greatly different than those in the sFOV (CT numberwater = −244 vs. CT number_water_ = 0), we did not evaluate the eFOV further.

### 3.2 Image distortion and volume changes

**Fig 2:**
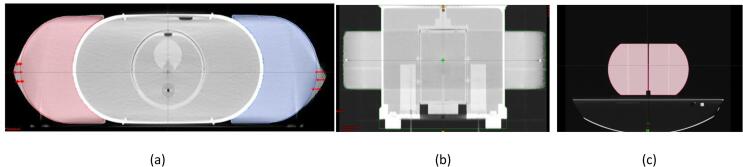
(a) Axial view of the pelvis phantom image with extended body parts attached. The double-headed red arrow indicates the imaging volume that extends beyond the actual volume (indicated by red and blue lines). (b) Coronal view of the body contour (displayed in green) extends outside the actual physical border of the phantom, as indicated by right/left BBs. The measured distance between left and right BBs is 60.77 cm versus the standard distance of 60 cm. (c) The extended body parts were imaged in standard field of view (sFOV) to compare differences in volume when the image was not distorted.

The extended body part volumes in the HD FOV were distorted from a half-circle to a more oval shape on each side (Fig. 2a). The measured distance between the left and right BBs was 60.77 cm versus the baseline distance of 60 cm (Fig. 2b). The mean measured volumes with the HD FOV were 3303 cm^3^ for the pelvis and 2671 cm^3^ for the lung, as compared with 3223 cm^3^ pelvis and 2487.5 cm^3^ lung in sFOV mode (Fig. 2c and Table 1), indicating a volume increase of 2.5% for the pelvis and 7.5% for the thorax phantoms when using HD FOV mode. The mean CT numbers of the acrylic parts were 39 (pelvis) and 32 (thorax) on HD FOV compared with standard mean CT numbers of 115 for acrylic material on sFOV mode, translating to decreases in CT numbers of –73 HU for pelvis and –87 HU for lung.

**Table 1:** Volumes and CT numbers (Hounsfield units) of acrylic body-part phantoms placed on either side of anthropomorphic prostate or lung phantoms.

	Rt HD FOV	Rt sFOV	Differences	Lt HD FOV	Lt sFOV	Difference
*Prostate phantom*						
Volume, cm^3^	3252	3200	**52**	3354	3246	**108**
Mean HU	46	113	**67**	32	111	**79**

*Lung phantom*						
Volume, cm^3^	2654	2487	**167**	2688	2488	**200**
Mean HU	41	119	**78**	23	119	**96**

Abbreviations: Rt, right; Lt, left; HD, high-definition; FOV, field of view; sFOV, standard scanning field of view.

### 3.3 Image quality

**Fig 3:**
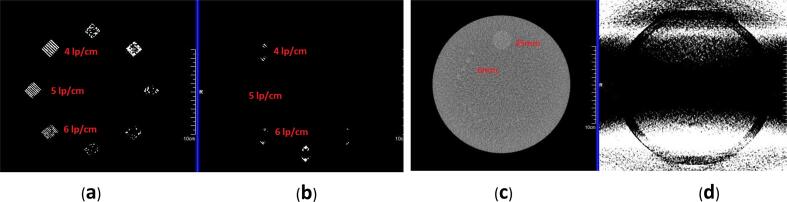
Comparison of high- and low-contrast resolution inserts in the American College of Radiology (ACR) phantom. (a) High-contrast line pair using standard field of view (sFOV). The image can resolve 6 line pairs per cm (lp/cm) object. (b) Image using the high-definition (HD) FOV option. Image of HD FOV cannot resolve a 4 lp/cm object. Both images were viewed with window = 100 and level = 1100. (c) Low-contrast section image comparison. The image in sFOV can resolve 6-mm hole objects. (d) Shadow artifacts and increased noise level prevented the resolution of 25-mm low-contrast objects imaged in HD FOV. Both images were viewed with standard viewing conditions for low-contrast objects (window = 100, level = 100).

High-contrast resolution was found to be reduced from 6 lp/cm in sFOV mode to none in HD FOV mode (Fig. 3a, b), when both images were viewed under the ACR-recommended standard viewing conditions for high-contrast subjects (i.e., window = 100 and level = 1100). Low-contrast resolution was also degraded; for a 0.6%-contrast object, the sFOV image without extended body parts truncated in the HD FOV and eFOV regions could resolve a 6-mm diameter, whereas the HD FOV mode with extended body parts truncated resolved nothing under standard viewing conditions (i.e., window = 100 and level = 100) (Fig. 3c,d).

### 3.4 Dosimetric accuracy

The TLD doses measurements were within ±3% agreement with the doses reported by the treatment planning system for all PTVs (Table 2). Dose agreement in the organs at risk (OARs) were also well within the IROC-established passing criteria (7% of average PTV dose) [13]. Film profiles through the center of the target were scaled to TLD dose values. A representative film profile that compares treatment planning values and film measurement of a prostate plan is shown in Supplementary Fig. S5. The film was subjected to gamma index analysis at the area of interest; the IROC passing criteria were 7% and 5 mm for the thorax and 7% and 4 mm for the pelvis. The pass rates for the gamma indexes were >97% for both phantoms (Table 3).

**Table 2:** Summary of thermoluminescence dosimetry results.

Location PTV	TPS–Reported Dose, Gy HD (sFOV)	TLD Dose, Gy HD (sFOV)	TLD/TPS HD (sFOV)	IROC Acceptability Criteria (TLD/TPS)
*Prostate Phantom*				
Prostate PTV (left)	6.15 (6.16)	6.33 (6.34)	1.03 (1.03)	0.93–1.07
Prostate PTV (right)	6.15 (6.16)	6.34 (6.44)	1.03 (1.05)	0.93–1.07

*Lung Phantom*				
Lung PTV (superior)	6.17 (6.27)	6.01 (6.33)	0.97 (1.01)	0.92–1.05
Lung PTV (inferior)	6.19 (6.23)	6.09 (6.25)	0.98 (1.00)	0.92–1.05

Location OAR	TPS–Reported Dose, Gy HD (sFOV)	TLD Dose, Gy HD (sFOV)	TLD/TPS HD (sFOV)	IROC Acceptability Criteria*, Gy HD (sFOV)
*Prostate Phantom*				
Femoral head (left)	0.93 (1.76)	1.09 (1.83)	—	0.65–1.54 (1.39–2.28)
Femoral head (right)	1.14 (1.57)	1.20 (1.64)	—	0.76–1.64 (1.19–2.09)

*Lung phantom*				
Spinal cord	1.34 (1.02)	1.26 (1.03)	—	0.83–1.68 (0.59–1.47)
Heart	0.97 (0.85)	0.77 (0.86)	—	0.35–1.19 (0.42–1.30)

*The ±7% criterion for the dose to the TLD located outside the target is quoted as a percentage of the average of PTV TLD doses and not as a local point.

**Table 3:** Summary film results. The spatial precision of the film and densitometer system was ±1 mm.

Film Plane	Gamma Index*	IROC Acceptability Criteria
Prostate PTV Coronal	98%	≥85%
Prostate PTV Sagittal	97%	≥85%
Lung PTV Axial	99%	≥80%
Lung PTV Coronal	97%	≥80%
Lung PTV Sagittal	97%	≥80%

*Percentage of points meeting the gamma-index criteria of 7% and 5 mm for lung, and 7% and 4 mm for prostate.

Abbreviations: IROC, Imaging and Radiation Oncology Core; PTV, planning target volume.

## 4 Discussion

Our test results indicate that with the CT scanner used for this work, the dosimetric accuracy of the treatment plans created using the CT images in HD FOV mode met the IROC passing criteria. As expected, CT numbers were different in the HD FOV region, with the difference ranging from 314 HU to 725 HU relative to those measured in the sFOV. These results exceed the manufacturer’s reported range of ±50 HU. However, the dosimetric effect of these changes was not large enough to have relevant clinical effects.

Perhaps because of the image reconstruction algorithm used by the manufacturer, the body-part volumes appeared larger in the HD FOV region, but the mean CT numbers decreased. These two factors seem to cancel each other out and result in negligible effects on the overall dose received by the PTVs and OARs. As for the volume change and its effects on the dose-volume histogram, we believe that most of the body parts extending into this region would be arms, shoulders, couch or immobilization devices that may not be of clinical interest. Our findings are consistent with results from the treatment planning perspective by Cheung et al. [6]. However, the presence of implants could lead to increased dose uncertainty that must be evaluated carefully. Also, a previous study of a large-bore CT scanner [7] showed that CT number distortions can be up to –356 HU for bone tissue and up to 323 HU for lung tissue, leading to contour distortion. However, in dosimetric terms, this inaccuracy is not relevant, as the calculated reduction in tumor dose was only about 3.0% of the target dose [7]. Results from the current study are consistent with these findings. Another group used anthropomorphic phantoms to study image distortion and CT number accuracy of a wide-bore CT scanner, and found similar results [8]. As suggested by a previous study [19], the dosimetric accuracy of treatment plans can reasonably be assumed to be within ±4% of target dose for all treatment sites in a worst-case scenario if the CT number variations are within ±100 HU of its standard value. As for the dense bone material, we hypothesize that the dose deviation delivered to the targets would not have clinically relevant effects, although future studies of this issue are needed. Moreover, we found that CT numbers in the eFOV region were off substantially compared with the sFOV option, and thus no further quantitative evaluation was justified. The manufacturer specifies visualization only in this region.

Although image distortion has been reported [7], no attempts have been made to quantify the effects of such distortion on image quality (high-contrast and low-contrast resolution) within the sFOV resulting from the truncation effect when body parts extend beyond the sFOV. Our results imply that imaging quality also suffers inside the sFOV when body parts extend beyond the HD FOV or eFOV regions, which may reduce the accuracy of target delineation. Therefore, care should be taken during the contouring process for low-contrast targets or organs (e.g., CTVs and prostate and parotid glands) whenever HD FOV is used.

Notably, however, changes in dose measurements were larger for OARs than for PTVs, because the dose gradient in the region between the OAR and the primary PTV can be quite steep, and any small displacement will result in a large difference in the dose delivered to the OAR. The IROC has set the dose agreement evaluation criteria for OARs to be ±7% of the PTV dose [14]. Our testing results were well within these limits. Nevertheless, the IROC passing criteria were set bearing various uncertainties in mind [13], such as small field size, treatment modality, modeling of multi-leaf collimators, and human error. The use of the HD FOV option should be minimized to improve plan robustness. This can be accomplished by paying close attention to centering patients and to the couch height during CT scanning to encompass all body parts into the sFOV to reduce uncertainty.

Our study did have some limitations. The extended body parts that we constructed represent a limited scope of patient setup scenariaos. The degree of such extension outside the sFOV and the HD FOV could be more significant in, say, breast simulation. Futher studies of such extension are needed for the sFOV as well. Moreover, deviations in measured dose result from not only patient geometry or FOV but also from other sources (e.g., those related to translation from CT numbers into mass density, beam models, and others). Further, our comparison of measured dose and planned dose in the phantom without the body parts revealed that they were still within the IROC-specified limits (Table 2). Finally, our tests were limited to photon therapy. Proton therapy is increasingly being used worldwide [20], [21], and many proton therapy center have large-bore CT simulators. We did not evaluate the accuracy of proton doses and ranges using the HD FOV mode, but this topic is also worthy of study in future work.

In conclusion, this large bore CT scanner with a 65-cm HD FOV mode is adequate for radiation oncology treatment simulation. The dosimetry results met or exceeded the IROC standard for accreditation. Image resolution was reduced by the use of HD FOV, which may affect the accuracy of contouring.

## Declaration of Competing Interest

The authors declare the following financial interests/personal relationships which may be considered as potential competing interests: Dr. Frank reports personal fees from Varian, grants and personal fees from C4 Imaging, grants from Eli Lily, grants from Elekta, grants and personal fees from Hitachi, other from Breakthrough Chronic Care, personal fees and other from Augmenix/Boston Scientific, personal fees from National Comprehensive Cancer Center (NCCN), outside the submitted work. Other authors have none.

## Funding

Supported in part by Cancer Center Support (Core) Grant CA016672 from the 10.13039/100000054National Cancer Institute, 10.13039/100000002National Institutes of Health, to 10.13039/100007313The University of Texas MD Anderson Cancer Center.
