# Supplementary material for: Evaluation of the high definition field of view option of a large-bore computed tomography scanner for radiation therapy simulation
Source: Phys Imaging Radiat Oncol. 2020 Mar 26;13:44–9. doi: 10.1016/j.phro.2020.03.004 (PMC7302052; doi:10.1016/j.phro.2020.03.004)
Supplement: Supplementary data 1 [file mmc1.pptx]

## Slide 1
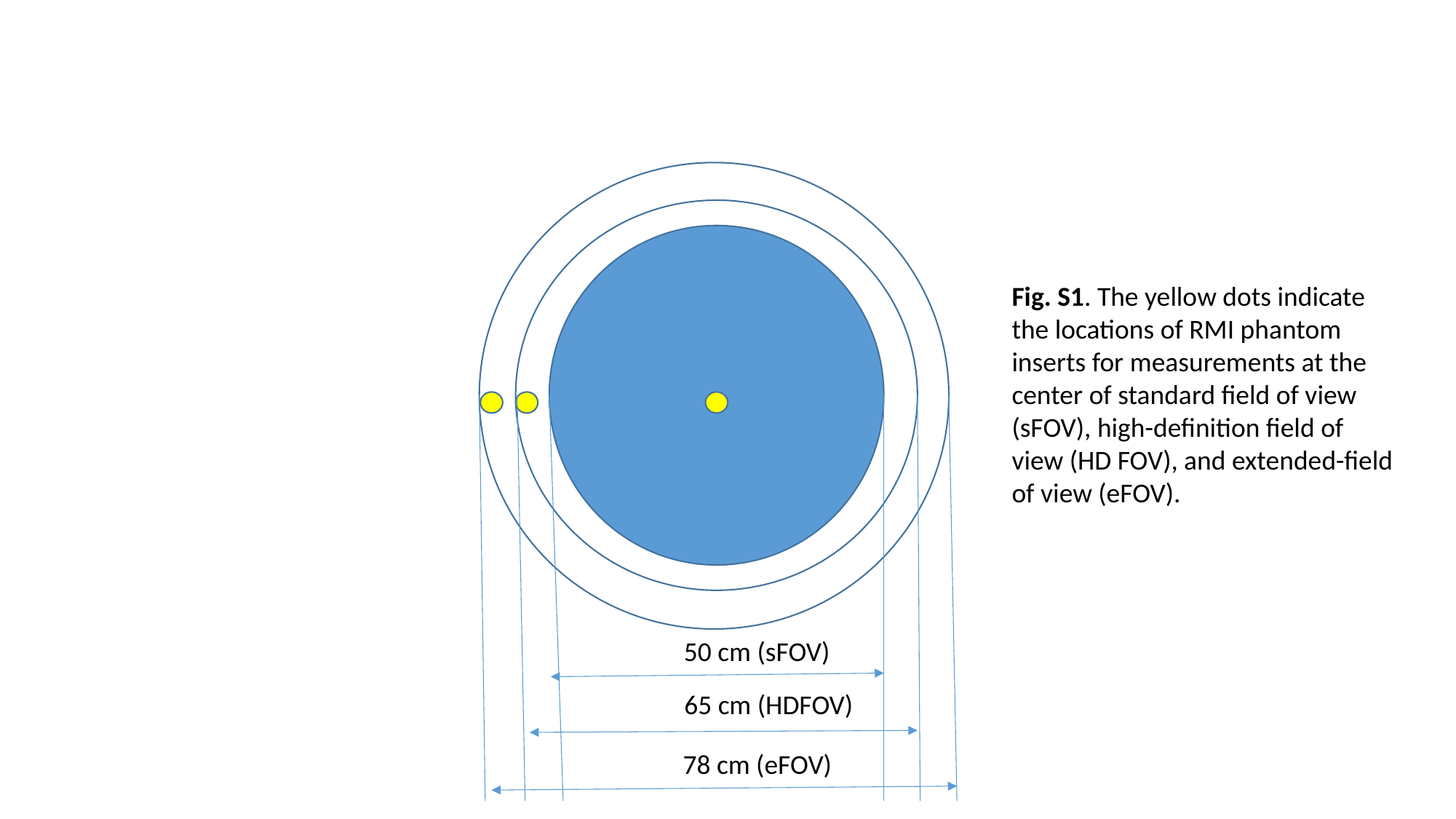

Fig. S1. The yellow dots indicate the locations of RMI phantom inserts for measurements at the center of standard field of view (sFOV), high-definition field of view (HD FOV), and extended-field of view (eFOV).
50 cm (sFOV)
65 cm (HDFOV)
78 cm (eFOV)

## Slide 2
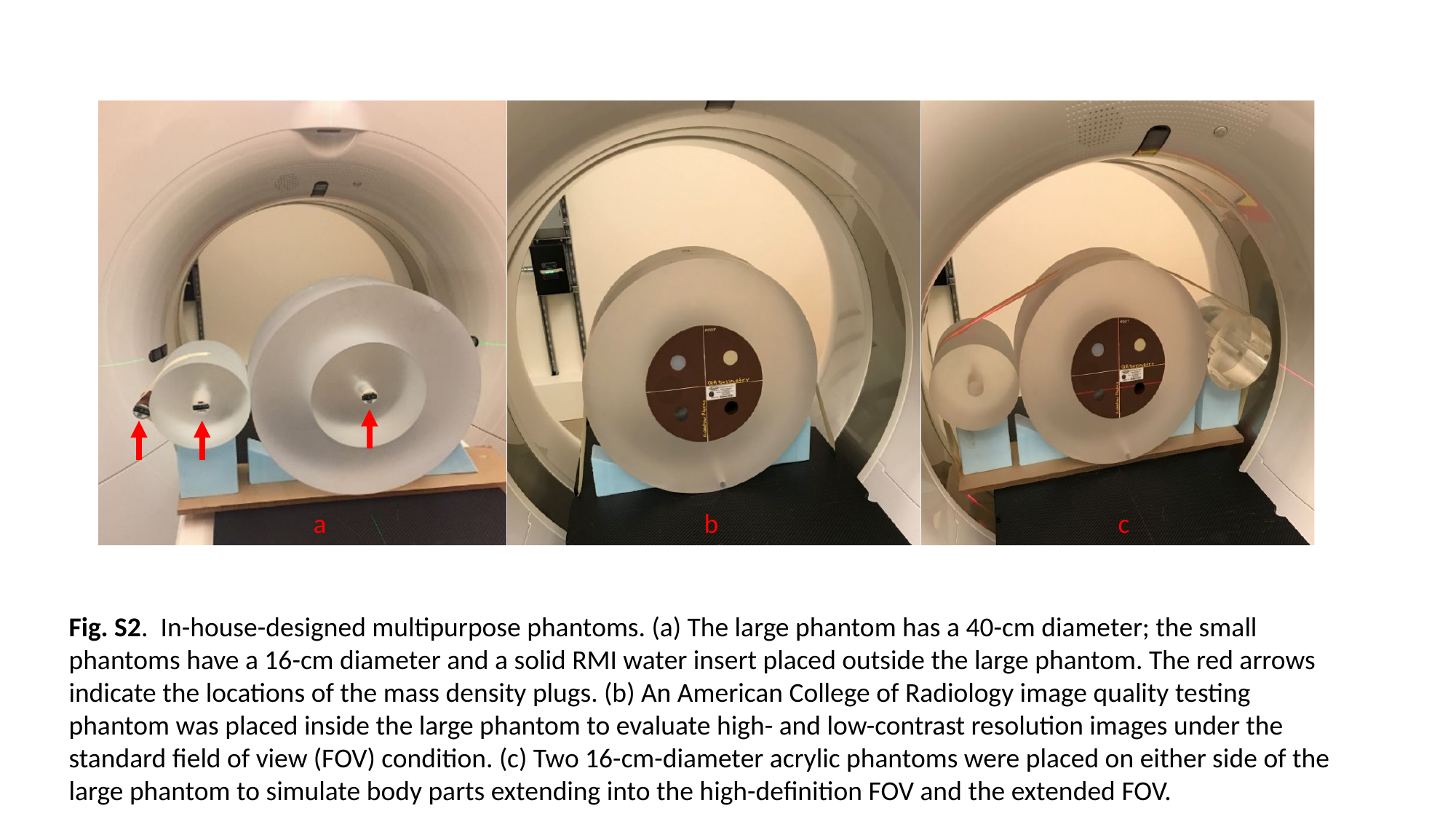

a
b
c
Fig. S2. In-house-designed multipurpose phantoms. (a) The large phantom has a 40-cm diameter; the small phantoms have a 16-cm diameter and a solid RMI water insert placed outside the large phantom. The red arrows indicate the locations of the mass density plugs. (b) An American College of Radiology image quality testing phantom was placed inside the large phantom to evaluate high- and low-contrast resolution images under the standard field of view (FOV) condition. (c) Two 16-cm-diameter acrylic phantoms were placed on either side of the large phantom to simulate body parts extending into the high-definition FOV and the extended FOV.

## Slide 3
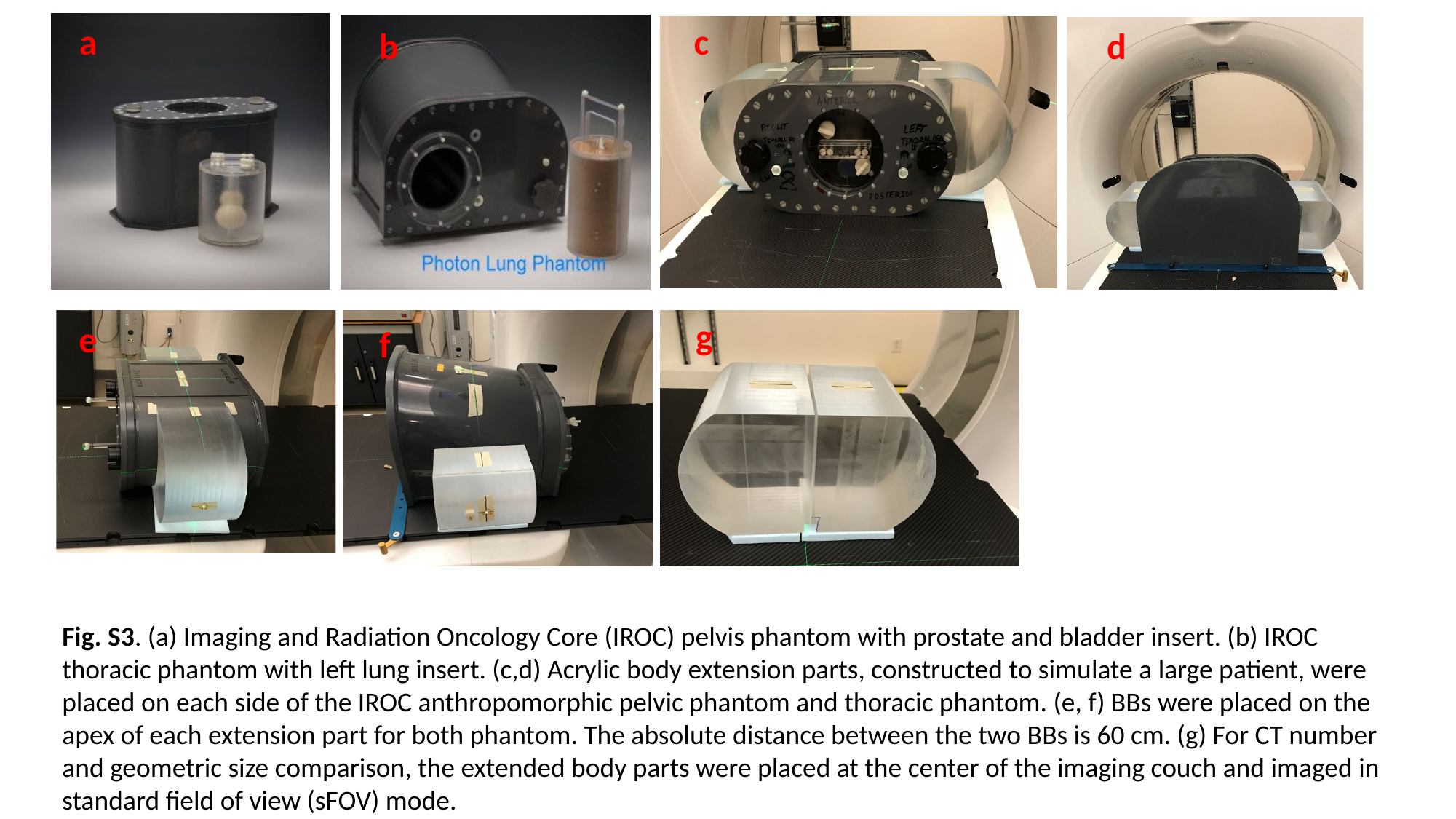

c
a
b
d
g
e
f
Fig. S3. (a) Imaging and Radiation Oncology Core (IROC) pelvis phantom with prostate and bladder insert. (b) IROC thoracic phantom with left lung insert. (c,d) Acrylic body extension parts, constructed to simulate a large patient, were placed on each side of the IROC anthropomorphic pelvic phantom and thoracic phantom. (e, f) BBs were placed on the apex of each extension part for both phantom. The absolute distance between the two BBs is 60 cm. (g) For CT number and geometric size comparison, the extended body parts were placed at the center of the imaging couch and imaged in standard field of view (sFOV) mode.

## Slide 4
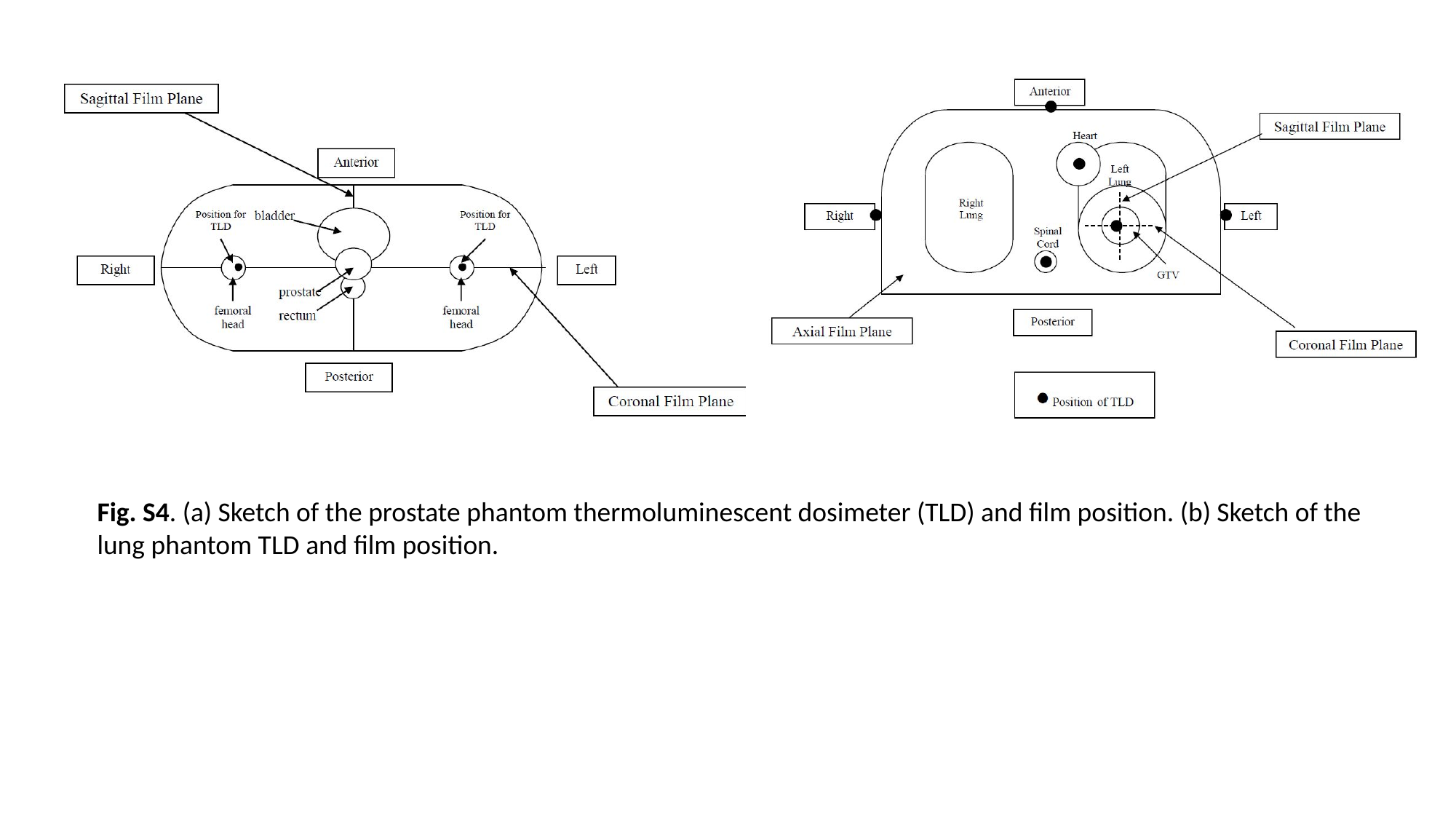

Fig. S4. (a) Sketch of the prostate phantom thermoluminescent dosimeter (TLD) and film position. (b) Sketch of the lung phantom TLD and film position.

## Slide 5
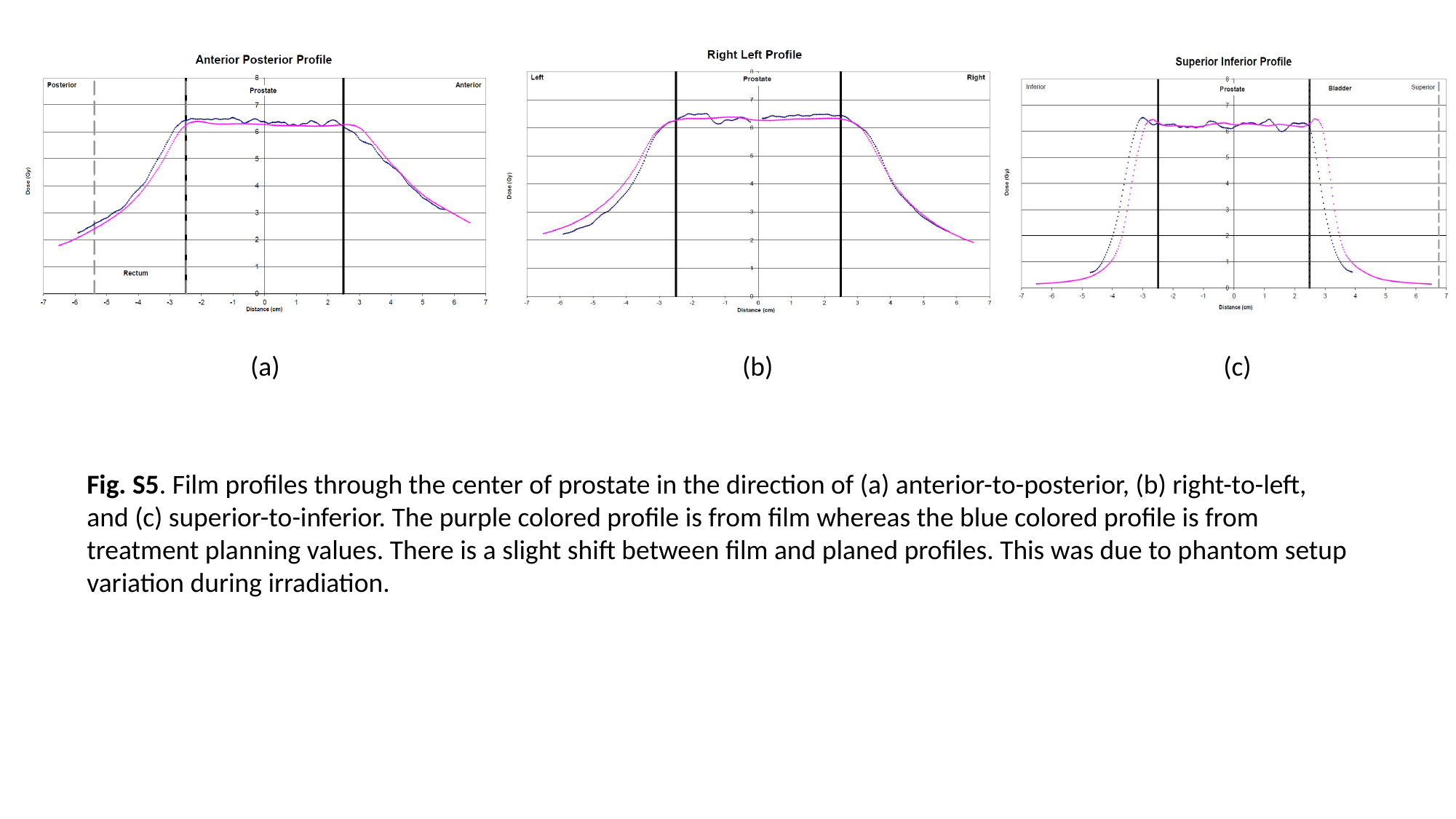

(a)
(b)
(c)
Fig. S5. Film profiles through the center of prostate in the direction of (a) anterior-to-posterior, (b) right-to-left, and (c) superior-to-inferior. The purple colored profile is from film whereas the blue colored profile is from treatment planning values. There is a slight shift between film and planed profiles. This was due to phantom setup variation during irradiation.
